# New synonyms of Tipula (Vestiplex) balioptera Loew, 1863 (Diptera, Tipulidae)

**DOI:** 10.3897/BDJ.13.e141363

**Published:** 2025-03-13

**Authors:** Pavel Starkevich, Jukka Salmela, Kjell Magne Olsen, Michael Andersson

**Affiliations:** 1 Nature Research Centre, Vilnius, Lithuania Nature Research Centre Vilnius Lithuania; 2 Regional Museum of Lapland, Rovaniemi, Finland Regional Museum of Lapland Rovaniemi Finland; 3 Biofokus, Oslo, Norway Biofokus Oslo Norway; 4 Independent Researcher, Huskvarna, Sweden Independent Researcher Huskvarna Sweden

**Keywords:** Distribution, hypopygium, species, taxonomy, Tipulinae

## Abstract

**Background:**

The Catalogue of Crane Flies of the World accounts for 196 recent species of subgenus Tipula (Vestiplex) Bezzi, 1924 distributed in Nearctic, Palaearctic and Oriental Regions (Oosterbroek 2024). The current paper provides taxonomic review of T. (V.) balioptera with designation of two new synonyms.

**New information:**

Tipula (Vestiplex) perretti Alexander, 1928 is designated as junior synonym of T. (V.) balioptera Loew, 1863. Tipula (V.) bo Mannheims, 1967 is removed from synonymy with T. (V.) tchukchi Alexander, 1934 and designated as the second junior synonym of T. (V.) balioptera. Tipula (V.) tchuckhi Alexander, 1934 is exluded from the list of fauna of Norway, Sweden and Finland and from West Palaearctic Region subsequently.

## Introduction

The Catalogue of Crane Flies of the World accounts for 196 recent species of subgenus Tipula (Vestiplex) Bezzi, 1924 distributed in Nearctic, Palaearctic and Oriental Regions (Oosterbroek 2024). Amongst them, 26 species were described as new to science from East Palaearctic and Oriental Regions in the last 8 years (Men et al. 2017, Starkevich et al. 2019a, Starkevich et al. 2019b, Men et al. 2021a, Men et al. 2021b, Men et al. 2021c, Ren et al. 2021, Yang et al. 2021 ,Men et al. 2021a, Men et al. 2021b, Men et al. 2021c ,Men et al. 2023, Starkevich and Young 2023). The current paper provides taxonomic review of T. (V.) balioptera with designation of two new synonyms.

## Materials and methods

Abbreviations for institutional collections used herein:

- ANSP = the Academy of Natural Sciences of Drexel University, USA;
- CNC = the Canadian National Collection of Insects, Arachnids and Nematodes, Ottawa, Canada;
- LMM = Regional Museum of Lapland, Rovaniemi, Finland;
- MCZ = Museum of Comparative Zoology, Harvard University, Cambridge, Massachusetts, USA;
- PCKMO = Private reference collection of Kjell Magne Olsen, Oslo, Norway;
- TROM = The Arctic University Museum of Norway, Tromsø, Norway;
- USNM = United States National Museum of Natural History, Washington, D.C., USA;
- MZLU = Lund University Biological Museum, Lund, Sweden.

For morphological analysis, terminalia of adult crane flies were removed and boiled in 10% sodium hydroxide (NaOH) for 5–10 minutes and observed in glycerine under an Olympus SZX10 stereomicroscope (Olympus, Japan). Photographs of adult specimens were taken with a Canon EOS 6D camera and Canon MP-E65 mm lens using the MJKZZ automated focus stacking rail set. Pictures of dissected terminalia were taken with an Infinity1 camera mounted on a Nikon Si-L stereomicroscope. The obtained layers were stacked using the programme ZereneStacker and edited with Photoshop. The Norwegian specimen of T. (V.) balioptera was imaged by using a Leica M205C stereomicroscope, Flexacam C1 camera and LAS X software. Digital images were stacked by using Helicon Focus software. Swedish specimens of T. (V.) bo were imaged by using a Canon EOS R and Canon MP-E65 mm lens. A stack was obtained by using Cognisys Stackshot 3X automated focus stacking rail and was processed with Helicon Focus 8 and edited in Photoshop.

Descriptive terminology of the adults generally follows that of Cumming and Wood (2017). The term genital bridge was adopted from Dobrotworsky (1968) which is equal to sclerites sp1 and sp2 (Neumann 1958).

## Taxon treatments

### Tipula (Vestiplex) balioptera

Loew, 1863

D43236A7-A3F4-5CBD-A981-66D9DAD5765E

https://v3.boldsystems.org/index.php/Public_RecordView?processid=FINTI086-11

*Tipulabalioptera* Loew, 1863 - Loew 1863: 284; Alexander 1915: 460;
Tipula (Vestiplex) balioptera: Crampton et al. 1942, Alexander 1965, Starkevich and Paramonov 2016, Starkevich et al. 2020, Brodo et al. 2023, Kolcsár et al. 2023;
= Tipula (Vestiplex) laccata laccata: Savchenko 1964 nec Lundstrom and Frey 1916 (partim), after Starkevich and Paramonov (2016);
= Tipula (Vestiplex) tchukchi: Mannheims 1971 nec Alexander 1934 (partim), after Starkevich and Paramonov (2016). According to Mannheims (1971), T. (V.) tchukchi = T. (V.) laccata: Lackschewitz 1936 (partim); Mannheims 1953 (partim); Savchenko 1964 (partim); nec Lundstrom and Frey 1916;
= Tipula (Vestiplex) tchukchi:Salmela 2008, Salmela 2011, Salmela 2012, Salmela and Petrašiūnas 2014;
Tipula (Vestiplex) bo Mannheims, 1967 - Mannheims 1967: 148 **syn. nov. stat. res.** Synonymy with T. (V.) tchukchi Alexander, 1934 after Mannheims and Savchenko (1973) = *laccata* Lackschewitz 1936 (partim), Mannheims 1953 (partim), Savchenko 1964 (partim), nec Lundstrom and Frey 1916;
*Tipulaperretti* Alexander, 1928 - Alexander 1928: 98 **syn. nov.**; Tipula (Vestiplex) perretti: Crampton et al. 1942.

#### Materials

- **Type status:** Lectotype. **Occurrence:** recordedBy: Kennicot; individualCount: 1; sex: male; **Taxon:** scientificName: Tipula (Vestiplex) balioptera Loew, 1863; **Location:** country: Canada; verbatimLocality: English River; **Record Level:** institutionCode: MCZ
- **Type status:** Other material. **Occurrence:** recordedBy: I. Kakko; individualCount: 2; sex: male, female; **Taxon:** scientificName: Tipula (Vestiplex) balioptera Loew, 1863; **Location:** country: Finland; verbatimLocality: Enontekiö, Bombovarri SE; **Event:** verbatimEventDate: 15 VII 2010; **Record Level:** institutionCode: LMM; source: http://tun.fi/NVO.TIPU391
- **Type status:** Other material. **Occurrence:** recordedBy: I. Kakko; individualCount: 2; sex: male; **Taxon:** scientificName: Tipula (Vestiplex) balioptera Loew, 1863; **Location:** country: Finland; verbatimLocality: Enontekiö, Bombovarri SE; **Event:** verbatimEventDate: 15 VII 2010; **Record Level:** institutionCode: LMM; source: http://tun.fi/NVO.TIPU392
- **Type status:** Other material. **Occurrence:** recordedBy: A. Haarto; individualCount: 1; sex: male; **Taxon:** scientificName: Tipula (Vestiplex) balioptera Loew, 1863; **Location:** country: Finland; verbatimLocality: Enontekiö, Urtasvaara; **Event:** verbatimEventDate: 15 VII 2009; **Record Level:** institutionCode: LMM; source: http://tun.fi/NVO.TIPU408
- **Type status:** Other material. **Occurrence:** recordedBy: J. Salmela; individualCount: 1; sex: male; **Taxon:** scientificName: Tipula (Vestiplex) balioptera Loew, 1863; **Location:** country: Finland; verbatimLocality: Enontekiö, Tomuttioja; **Event:** verbatimEventDate: 11 VI – 19 VII 2009; **Record Level:** institutionCode: LMM; source: http://tun.fi/NVO.TIPU824
- **Type status:** Other material. **Occurrence:** recordedBy: J. Salmela; individualCount: 1; sex: female; associatedSequences: BOLD sample ID JES-20110086; **Taxon:** scientificName: Tipula (Vestiplex) balioptera Loew, 1863; **Location:** country: Finland; verbatimLocality: Enontekiö, Pahtavaara; **Event:** verbatimEventDate: 11 VI – 19 VII 2009; **Record Level:** institutionCode: LMM; source: http://tun.fi/NVO.TIPU1858
- **Type status:** Other material. **Occurrence:** recordedBy: J. Salmela; individualCount: 1; sex: male; associatedSequences: BOLD sample ID JES-20110129; **Taxon:** scientificName: Tipula (Vestiplex) balioptera Loew, 1863; **Location:** country: Finland; verbatimLocality: Enontekiö, Tomuttioja; **Event:** verbatimEventDate: 11 VI – 19 VII 2009; **Record Level:** institutionCode: LMM; source: http://tun.fi/NVO.TIPU1896
- **Type status:** Other material. **Occurrence:** recordedBy: J. Salmela; individualCount: 1; sex: male; **Taxon:** scientificName: Tipula (Vestiplex) balioptera Loew, 1863; **Location:** country: Finland; verbatimLocality: Utsjoki, Galddasjohka; **Event:** verbatimEventDate: 18 VI – 19 VII 2007; **Record Level:** institutionCode: LMM; source: http://tun.fi/NVO.TIPU825
- **Type status:** Other material. **Occurrence:** recordedBy: A. Rinne; individualCount: 1; sex: male; **Taxon:** scientificName: Tipula (Vestiplex) balioptera Loew, 1863; **Location:** country: Finland; verbatimLocality: Utsjoki, Teno; **Event:** verbatimEventDate: 22 VI – 3 VII 2006; **Record Level:** institutionCode: LMM; source: http://tun.fi/NVO.TIPU851
- **Type status:** Other material. **Occurrence:** recordedBy: A. Järvinen; individualCount: 1; sex: male; **Taxon:** scientificName: Tipula (Vestiplex) balioptera Loew, 1863; **Location:** country: Norway; verbatimLocality: Troms, Storfjord, Skibotn, Veajetnibba S; **Event:** verbatimEventDate: 24 VI – 16 VII 2017; **Record Level:** institutionCode: LMM; source: http://tun.fi/NVO.ins2018-168
- **Type status:** Other material. **Occurrence:** recordedBy: A. Järvinen; individualCount: 1; sex: male; occurrenceID: 8016767E-E022-557C-BF0F-F718132F850F; **Taxon:** scientificName: Tipula (Vestiplex) balioptera Loew, 1863; **Location:** country: Norway; verbatimLocality: Veajetnibba S; **Event:** verbatimEventDate: 19 VII – 12 VIII 2017; **Record Level:** institutionCode: LMM; source: http://tun.fi/NVO.ins2018-199
- **Type status:** Other material. **Occurrence:** recordedBy: A. Järvinen; individualCount: 3; sex: 2 males, 1 female; **Taxon:** scientificName: Tipula (Vestiplex) balioptera Loew, 1863; **Location:** country: Norway; verbatimLocality: Veajetnibba S; **Event:** verbatimEventDate: 16 VII – 19 VII 2017; **Record Level:** institutionCode: LMM; source: http://tun.fi/NVO.ins2018-205
- **Type status:** Other material. **Occurrence:** catalogNumber: 721756; recordedBy: K.M. Olsen; individualCount: 1; sex: male; occurrenceID: F7C421F7-3612-5A86-8672-1B18AE8B97C9; **Taxon:** scientificName: Tipula (Vestiplex) balioptera Loew, 1863; **Location:** country: Norway; verbatimLocality: Troms, Kåfjord, N Nuorrtit Gussačohkka; **Event:** verbatimEventDate: 12 VII 2022; **Record Level:** institutionCode: PCKMO
- **Type status:** Other material. **Occurrence:** catalogNumber: 718592; recordedBy: K.M. Olsen; individualCount: 5; sex: 3 males, 2 females; occurrenceID: 6797498A-FFE2-5196-A7AB-00AA10C38760; **Taxon:** scientificName: Tipula (Vestiplex) balioptera Loew, 1863; **Location:** country: Norway; verbatimLocality: N Nuorrtit Gussačohkka; **Event:** verbatimEventDate: 1 VII – 12 VII 2022; **Record Level:** institutionCode: PCKMO
- **Type status:** Other material. **Occurrence:** catalogNumber: TROM TSZD 18002; recordedBy: T. Soot-Ryen; individualCount: 1; sex: male; **Taxon:** scientificName: Tipula (Vestiplex) balioptera Loew, 1863; **Location:** country: Norway; verbatimLocality: Finnmark, Alta, Jotkajavre; **Event:** verbatimEventDate: 3 VII 1924; **Record Level:** institutionCode: TROM
- **Type status:** Other material. **Occurrence:** catalogNumber: TROM TSZD 18001; recordedBy: T. Soot-Ryen; individualCount: 1; sex: male; **Taxon:** scientificName: Tipula (Vestiplex) balioptera Loew, 1863; **Location:** country: Norway; verbatimLocality: Finnmark, Alta, Jotkajavre; **Event:** verbatimEventDate: 9 VII 1924; **Record Level:** institutionCode: TROM
- **Type status:** Other material. **Occurrence:** catalogNumber: TROM TSZD 18004; recordedBy: T. Soot-Ryen; individualCount: 1; sex: male; **Taxon:** scientificName: Tipula (Vestiplex) balioptera Loew, 1863; **Location:** country: Norway; verbatimLocality: Finnmark, Alta, Jotkajavre; **Event:** verbatimEventDate: 9 VII 1924; **Record Level:** institutionCode: TROM
- **Type status:** Other material. **Occurrence:** catalogNumber: TROM TSZD 18006; recordedBy: T. Soot-Ryen; individualCount: 1; sex: female; **Taxon:** scientificName: Tipula (Vestiplex) balioptera Loew, 1863; **Location:** country: Norway; verbatimLocality: Finnmark, Alta, Jotkajavre; **Event:** verbatimEventDate: 9 VII 1924; **Record Level:** institutionCode: TROM
- **Type status:** Other material. **Occurrence:** catalogNumber: TROM TSZD 18003; recordedBy: T. Soot-Ryen; individualCount: 1; sex: male; **Taxon:** scientificName: Tipula (Vestiplex) balioptera Loew, 1863; **Location:** country: Norway; verbatimLocality: Finnmark, Alta, Jotkajavre; **Event:** verbatimEventDate: 15 VII 1924; **Record Level:** institutionCode: TROM
- **Type status:** Other material. **Occurrence:** catalogNumber: TROM TSZD 18005; recordedBy: T. Soot-Ryen; individualCount: 1; sex: male; **Taxon:** scientificName: Tipula (Vestiplex) balioptera Loew, 1863; **Location:** country: Norway; verbatimLocality: Finnmark, Alta, Bojobæski; **Event:** verbatimEventDate: 16 VII 1924; **Record Level:** institutionCode: TROM
- **Type status:** Holotype. **Occurrence:** catalogNumber: No. 2559; individualCount: 1; sex: male; preparations: Antenna and wing on slide mounted, USNM; **Taxon:** scientificName: Tipula (Vestiplex) perretti Alexander, 1928; **Location:** country: Canada; verbatimLocality: Hopedale, Labr; **Record Level:** institutionCode: CNC
- **Type status:** Paratype. **Occurrence:** catalogNumber: Type # 6365:2; MZLU-110421; recordedBy: A & S Ulfstrand; individualCount: 1; sex: male; **Taxon:** scientificName: Tipula (Vestiplex) bo Mannheims, 1967; **Location:** country: Sweden; verbatimLocality: Lycksele Lappmark, Vindelälven, N Tjatsemvare, 45 km NW Ammarnäs; **Event:** verbatimEventDate: 23 VII 1965; **Record Level:** institutionCode: MZLU; source: https://www.flickr.com/photos/tags/mzlutype06365/
- **Type status:** Paratype. **Occurrence:** catalogNumber: Type # 6365:3; MZLU-110422; recordedBy: A & S Ulfstrand; individualCount: 1; sex: male; **Taxon:** scientificName: Tipula (Vestiplex) bo Mannheims, 1967; **Location:** country: Sweden; verbatimLocality: Lycksele Lappmark, Vindelälven, N Tjatsemvare, 45 km NW Ammarnäs; **Event:** verbatimEventDate: 23 VII 1965; **Record Level:** institutionCode: MZLU; source: https://www.flickr.com/photos/tags/mzlutype06365/
- **Type status:** Paratype. **Occurrence:** catalogNumber: Type # 6365:4; MZLU-110423; recordedBy: A & S Ulfstrand; individualCount: 1; sex: female; **Taxon:** scientificName: Tipula (Vestiplex) bo Mannheims, 1967; **Location:** country: Sweden; verbatimLocality: Lycksele Lappmark, Vindelälven, N Tjatsemvare, 45 km NW Ammarnäs; **Event:** verbatimEventDate: 23 VII 1965; **Record Level:** institutionCode: MZLU; source: https://www.flickr.com/photos/tags/mzlutype06365/

#### Taxon discussion

The first record of T. (V.) balioptera out of the Nearctic Region was published by Starkevich and Paramonov (2016) with taxonomic account as follows:

= Tipula (Vestiplex) laccata laccata: Savchenko 1964 nec Lundstrom and Frey 1916 (partim), after Starkevich and Paramonov (2016).

A single male specimen from Yakutyia, Russia was detected amongst material identified as T. (V.) laccata by E. N. Savchenko (Savchenko 1964). Another partim of the T. (V.) laccata material belonged to T. (V.) aldrichiana, a species which was also recorded in the Palaearctic for the first time (Starkevich and Paramonov 2016). Later, T. (V.) balioptera was recorded in Mongolia (Starkevich et al. 2020) and Norway (Kolcsár et al. 2023).

Following Mannheims (1971), all material published by Savchenko (1964) as T. (V.) laccata was synonymised with T. (V.) tchukchi Alexander, 1934, including some other material listed below:

Tipula (Vestiplex) tchukchi Alexander, 1934

= *laccata* Lackschewitz 1936 (partim), Mannheims 1953 (partim), Savchenko 1964, nec Lundstrom and Frey 1916, after Mannheims (1971).

Finally, another species T. (V.) bo Mannheims, 1967 was synonymised by Mannheims and Savchenko (1973) with the taxonomic list as follows:

Tipula (Vestiplex) tchukchi Alexander, 1934

= *laccata* Lackschewitz 1936, Mannheims 1953, Savchenko 1964, nec Lundstrom and Frey 1916, after Mannheims and Savchenko (1973).

As can be seen from the taxonomic records (Mannheims 1971, Mannheims and Savchenko 1973), besides T. (V.) bo, three partims of T. (V.) laccata material were listed as T. (V.) tchukchi.

After examination of type material of T. (V.) bo and based on morphology analysis, we found that this species belongs to T. (V.) balioptera and thus is a junior synonym:

Tipula (Vestiplex) bo Mannheims, 1967 - Mannheims 1967: 148 **syn. nov. stat. res.**

= *laccata* Lackschewitz 1936 (partim), Mannheims 1953 (partim), Savchenko 1964 (partim), nec Lundstrom and Frey 1916.

Subsequently, T. (V.) tchukchi is exluded from the list of fauna from Norway, Sweden and Finland.

## Discussion

### Discussion on morphology results

**Figure 1.**
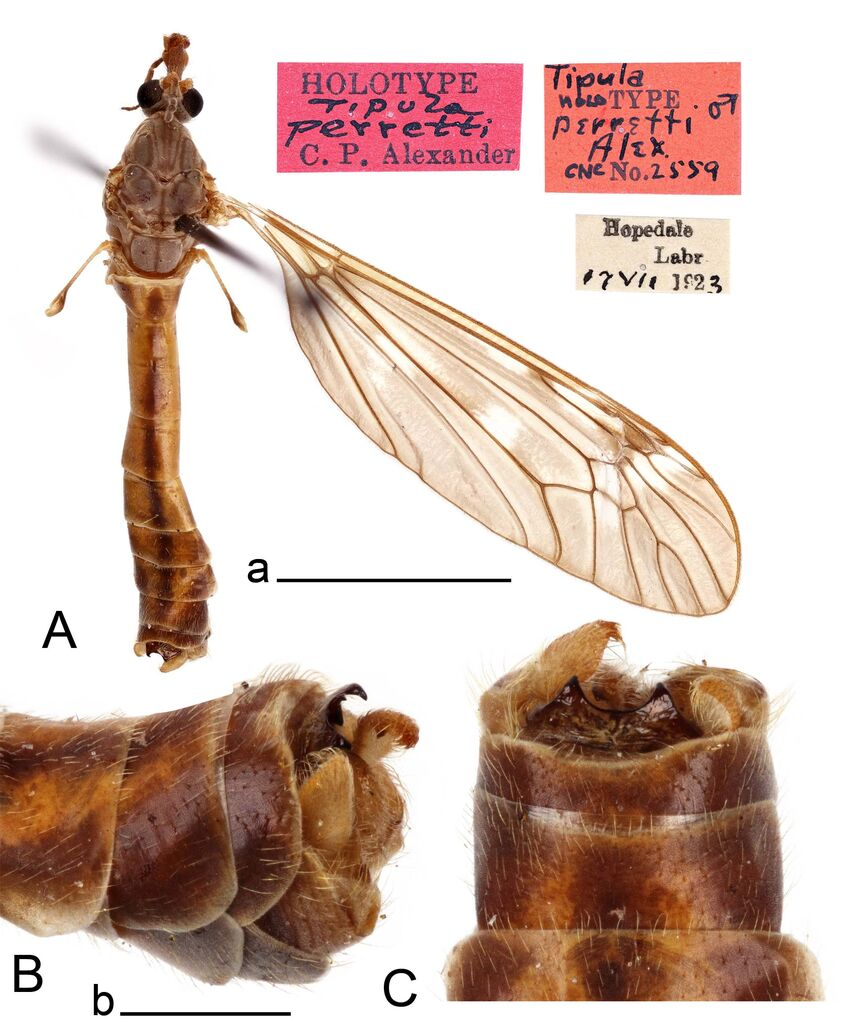
Male of Tipula (Vestiplex) perretti, holotype. **A** Habitus, dorsal view; **B** Hypopygium, lateral view; **C** Hypopygium, dorsal view. Scale bars: a (A) = 5 mm, b (B, C) = 1 mm.

**Figure 2.**
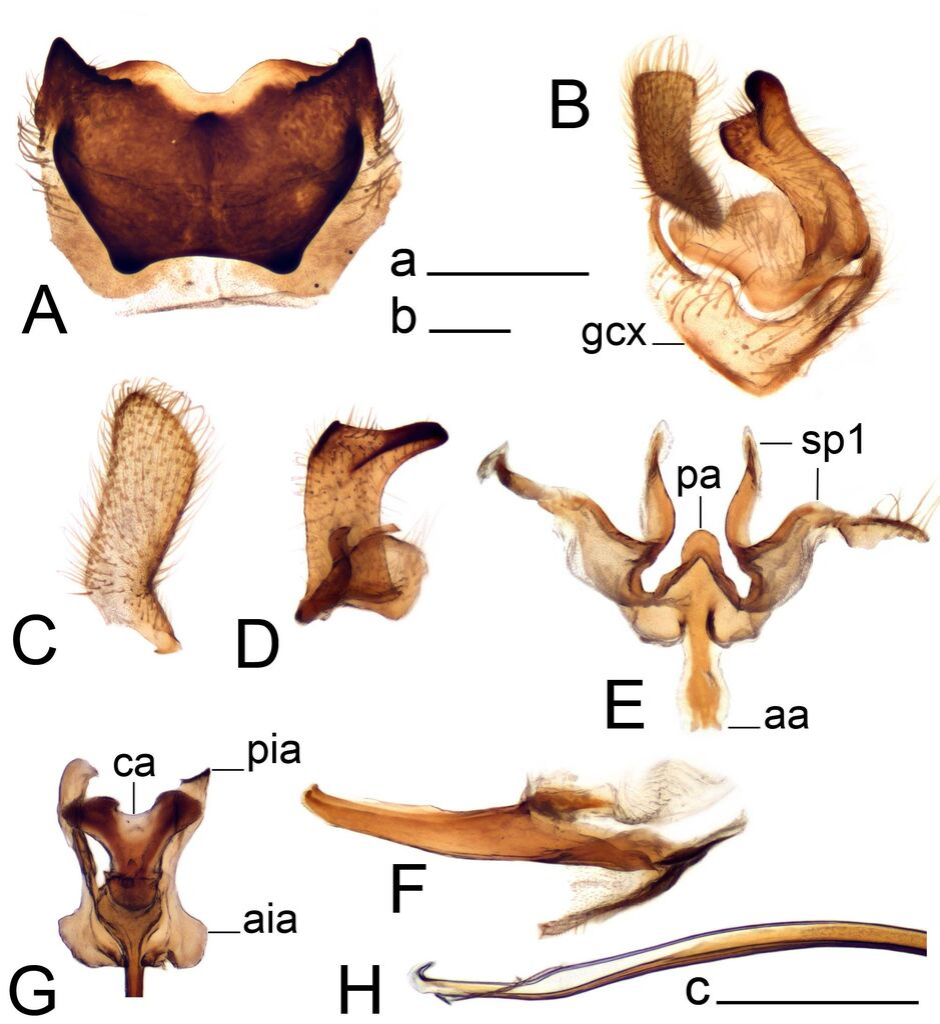
Hypopygium of male Tipula (Vestiplex) perretti, holotype. **A** Tergite 9, dorsal view; **B.** Left gonocoxite, outer and inner gonostyli, lateral view; **B.** Left outer gonostylus, lateral view; **C** Right outer gonostylus, lateral view; **D** Right inner gonostylus, lateral view; **E** Genital bridge, dorsal view; **F** Adminiculum, lateral view; **G** Sperm pump, dorsal view; **H** Distal part of aedeagus, lateral view. Abbreviations: aa, anterior apodeme; aia, anterior immovable apodeme; ca, compressor apodeme; gcx, gonocoxite; pa, posterior apodeme; pia, posterior immovable apodeme; sp1, sclerite sp1. Scale bars: a (A, C–G) = 0.5 mm, b (B) = 0.5 mm, c (H) = 0.25 mm.

**Figure 3.**
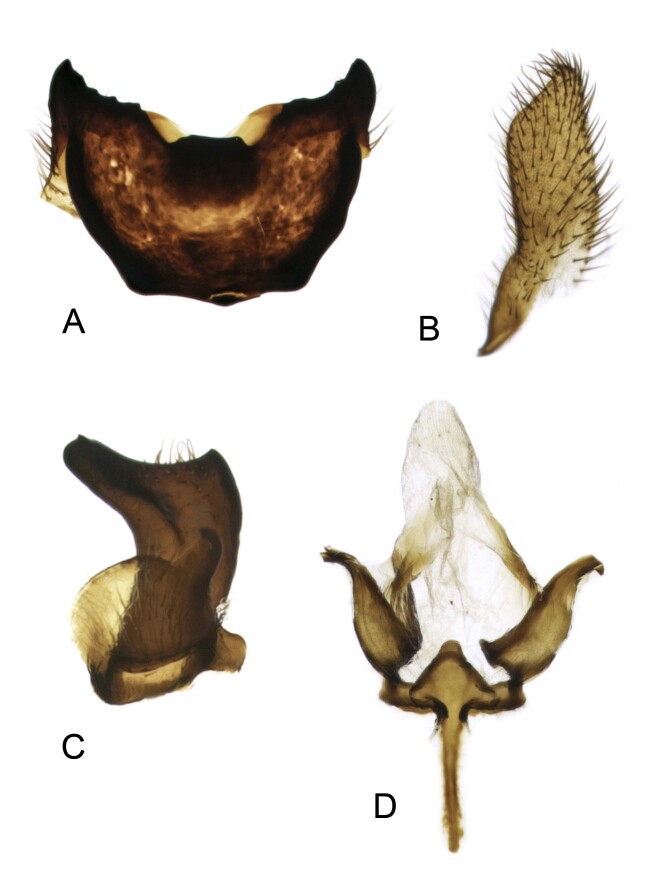
Hypopygium of male Tipula (Vestiplex) balioptera, Norway, NVO.ins2018-168. **A** Tergite 9, dorsal view; **B** Left outer gonostylus, lateral view; **C** Left inner gonostylus, lateral view; **D** Genital bridge with proctiger, dorsal view. Scale bar absent.

Tipula (V.) perretti was described from single male collected in Labrador, Canada by Alexander (1928). No additional material was published after the original description. The specimen was not dissected and limited drawing of male genitalia showing the caudal part of tergite 9 was only provided. In addition, the specific position of tergite 9 was omitted by Alexander, but described in his another work (Alexander 1934): “In a number of species of *Vestiplex*, the tergal saucer tends to swing ventrad so as to lie on the lower face of the tergite, in which case the only part of the sclerite visible from above is feebly sclerotized cephalic portion, together with the cephalic rim of the saucer, where this is developed. In such cases, when treated with caustic soda, the saucer swings dorsad and cephalad so as to assume its normal position. This curious occurrence was first noted by Mr. Edwards, and unless appreciated may cause confusion in separation of allied species”. The holotype of T. (V.) perretti represents described specific position of tergite 9 (Fig. 1B and C). In addition, inner gonostyli were also not visible due to the specific position of tergite 9. This possibly caused the issue with identification, so that the specimen was described as new species. After specimen examination and genitalia dissection, it turned out that the morphology is identical to that of T. (V.) balioptera, a species widely distributed throughout the Nearctic and Palaearctic (Oosterbroek 2024). The new record of T. (V.) balioptera in Europe stimulated revision of existing literature and material of local T. (Vestiplex) species and, as a consequence, the type material of T. (V.) bo was revised uncovering its identity with T. (V.) balioptera. Males of T. (V.) balioptera can be separated from its nearest ally T. (V.) laccata by details of tergite 9, outer and inner gonostyli. They can be also distinguished by the genital bride. Tergite 9 of T. (V.) balioptera posteriorly has a broad median notch, lateral angle extended, horn-shaped (Fig. 2A and Fig. 3A). In T. (V.) laccata, the posterior margin has a deep and narrow median notch and obtuse lateral angles (Starkevich et al. 2020: fig. 75).

Inner gonostylus of T. (V.) balioptera with beak extended (Fig. 2D and Fig. 3), while in T. (V.) laccata, beak is short (Starkevich et al. 2020: fig. 78). Genital bridge of T. (V.) balioptera with posterior apodeme triangular (Fig. 2E and Fig. 3D), while it is rounded in T. (V.) laccata (Starkevich et al. 2020: fig. 79). Females of T. (V.) balioptera can be easily separated by sternite 9 having additional lateral incisions (Starkevich et al. 2020: fig. 48) which are absent in T. (V.) laccata (Starkevich et al 2020: fig. 84).

Hypopygium. Tergite 9 forming a concave sclerotised saucer (Fig. 2A and Fig. 3A). Posterior margin of tergite 9 emarginated, with broad median V-shaped notch, with 2 yellow oblong projections and black median spinous tooth, additional small denticles on either side. Lateral angle of tergite 9 extended, horn-shaped. Anterior part of tergite 9 raised into sclerotised border, its lateral angle terminating into obtuse tooth. Gonocoxite unarmed (Fig. 2B). Outer gonostylus flattened, nearly parallel-sided with apex oblique (Fig. 2C and Fig. 3B). Inner gonostylus in shape of curved plate, dorsally with small acute spine, beak extended into rostrum with distal margin blackened, tipped with small tooth (Fig. 2D and Fig. 3C). Genital bridge with its sp1 forming anterior apodeme, posterior apodeme nearly triangular; sclerite sp1 well developed, bilobed (Fig. 2E and Fig. 3D). Aedeagal guide relatively narrow tube-shaped structure (Fig. 2F). Sperm pump with compressor apodeme having median incision (Fig. 2G). Posterior immovable apodeme slightly longer than compressor apodeme and slightly broadened. Anterior immovable apodeme irregularly-shaped. Aedeagus with distal part ventrally membranous, shovel-shaped (Fig. 2H).

**Figure 4.**
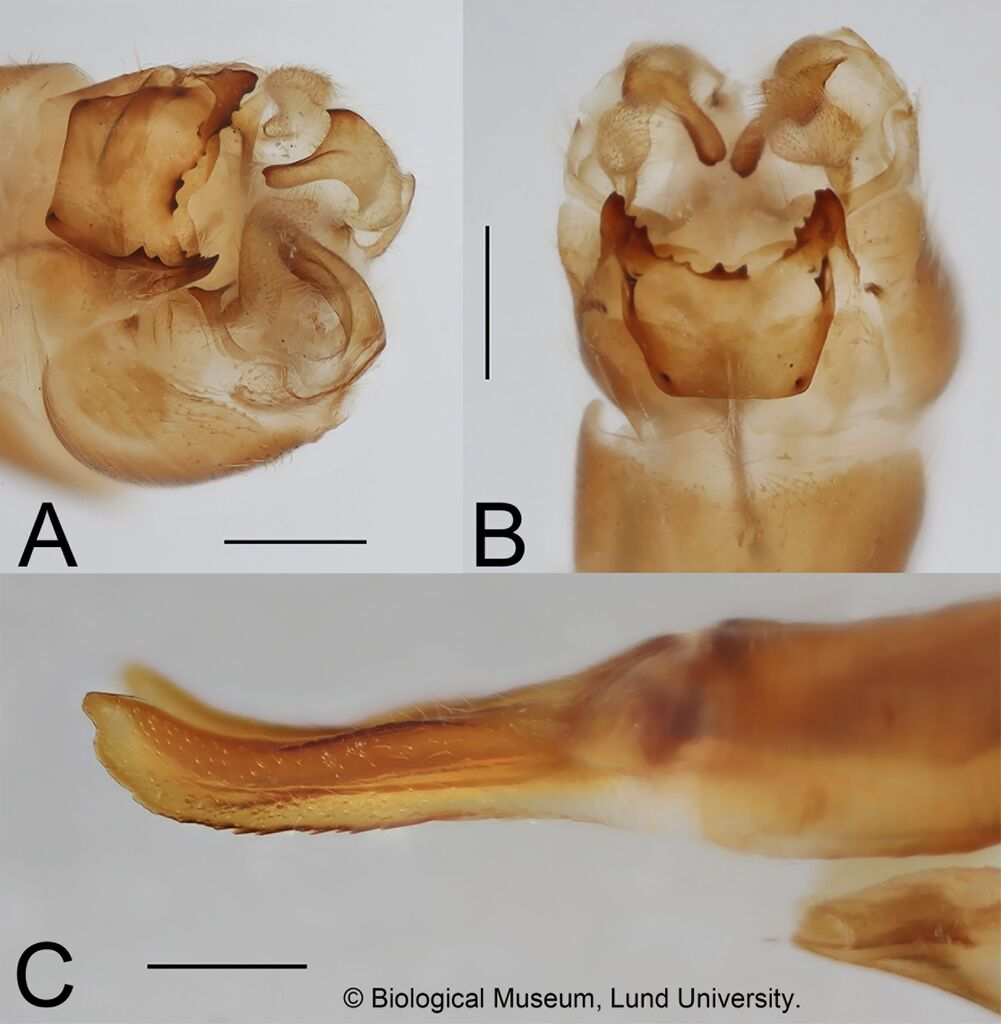
Tipula (Vestiplex) bo, male and female terminalia, paratypes. **A** Hypopygium dorso-lateral view; **B** Hypopygium dorsal view; **C** Ovipositor with cercus, lateral view. Scale bar = 0.5 mm.

Variation. The tergal median denticle on posterior margin of tergite 9 shows some variations, from single, obtuse (Fig. 2A) to stout (Fig. 3A) or split (Fig. 4A and B).

Ovipositor (Fig. 4C). Cercus yellowish, slightly shorter than tergite 10, with tip curved dorsally and with small incision (Fig. 2) which is absent in the Mongolian specimen; outer margin with rough and obtuse serration. Hypovalva in shape of pale elongated filament, lateral incision present (Starkevich et al. 2020: fig. 48).

## Supplementary Material

XML Treatment for Tipula (Vestiplex) balioptera
